# ASO Author Reflections: Improved Perioperative Seroma and Complication Rates Following the Application of a Two-Layer Negative Pressure Wound Therapy System After Inguinal Lymphadenectomy for Metastatic Cutaneous Melanoma

**DOI:** 10.1245/s10434-020-08525-3

**Published:** 2020-05-27

**Authors:** Marc Moncrieff, Martin Heaton

**Affiliations:** 1 Plastic and Reconstructive Surgery, Norfolk and Norwich University Hospital, Norwich, UK; 2 Faculty of Medicine and Health Sciences, Norwich Medical School, University of East Anglia, Norwich, UK

## Past

Inguinal lymphadenectomy is the current standard of care in the management of macroscopic nodal disease, but unfortunately postoperative complications are very common, especially seroma. Seroma rates from the published literature are, on average, approximately 50%.^1^ Multiple studies have investigated interventions designed to either reduce the incidence or reduce the extent of the problem once it has become established. A recent systematic review by the Cochrane Group^2^ identified a lack of any useful data and concluded that “… there is a need for high-quality randomized controlled trials to guide clinical practice in this under-researched area”. Seromas are important because they affect patients’ quality of life postoperatively, invariably cause patients to return repeatedly to the clinic over a period of many weeks for aspirations, and subsequent complications such as infection are common as a result.

## Present

In this retrospective study,^3^ we employed a two-layer negative pressure wound therapy system (2-LNPWT) as a method to reduce seroma rates and perioperative complications. We compared the outcomes of its use with traditional closed suction drains (CSDs). The key finding of this study was that there was a significant association with better postoperative outcomes using the 2-LNPWPT system in terms of incidence of seroma formation (26.9% vs. 49.4%; odds ratio [OR] 3.0; *p* < 0.03), period of drainage (15 days vs. 20 days; *p* = 0.005), and return to theater rate (0% vs. 15.3%; *p* = 0.03). When the factors for seroma formation were analyzed, the only significant association was the type of drainage system used (2-LNPWT: 31.2% vs. CSD: 58.3%; *p* < 0.03; OR 3.0 [1.1–8.3]). Furthermore, once a seroma was established, it was not possible to alter the clinical course of this complication, regardless of the drainage system that was originally employed. Finally, there was no significant difference in overall, disease-specific, or recurrence-free survival detected between the two groups.

## Future

This study^3^ has provided evidence that the incidence of seroma, and subsequent complications, can be significantly reduced with a 2-LNPWT system, regardless of disease burden or patient body mass index. Importantly, the data suggest that this is a safe technique in terms of disease-specific outcomes, despite previous theoretical concerns regarding promotion of tumor angiogenesis at the operation site.

The data indicate that a prospective randomized controlled trial is merited, with expansion to include other disease indications, particularly urological and gynaecological tumors. The utility of the technique in previously irradiated wounds would be of particular interest, although one would assume that the efficacy would be significantly diminished in these cases. With the current vogue towards endoscopic groin dissections,^4^ the technique would need further modification. In any case, given the relative ‘Heath Robinson’ design to our system, we would encourage the commercial sector to invest in product design to improve the facility for applying and changing the dressings in both the hospital and primary care settings.
